# Evaluation of the Impact of Low Emission Zone and Heavy Traffic Ban in Munich (Germany) on the Reduction of PM_10_ in Ambient Air

**DOI:** 10.3390/ijerph110505094

**Published:** 2014-05-13

**Authors:** Veronika Fensterer, Helmut Küchenhoff, Verena Maier, Heinz-Erich Wichmann, Susanne Breitner, Annette Peters, Jianwei Gu, Josef Cyrys

**Affiliations:** 1 Statistical Consulting Unit, Department of Statistics, Ludwig-Maximilians-Universität, Akademiestr. 1, Munich 80799, Germany; E-Mails: kuechenhoff@stat.uni-muenchen.de (H.K.); VeMaier@gmx.de (V.M.); 2 Helmholtz Zentrum München, Institute of Epidemiology I, Ingolstädter Landstr. 1, Neuherberg 85764, Germany; E-Mail: wichmann@helmholtz-muenchen.de; 3 Helmholtz Zentrum München, Institute of Epidemiology II, Ingolstädter Landstr. 1, Neuherberg 85764, Germany; E-Mails: susanne.breitner@helmholtz-muenchen.de (S.B.); peters@helmholtz-muenchen.de (A.P.); cyrys@helmholtz-muenchen.de (J.C.); 4 Environment Science Center, Universität Augsburg, Universitätsstr. 1a, Augsburg 86159, Germany; E-Mail: jianwei.gu@physik.uni-augsburg.de

**Keywords:** fine particles, PM_10_, low emission zone, truck ban, Munich

## Abstract

Concentrations of ambient fine particles (PM_10_: particles with an aerodynamic diameter ≤ 10 µm) are still exceeding current air quality standards in many European cities. In Munich (Germany), low emission zone and transit bans for heavy-duty vehicles were introduced in 2008 aiming at reduction of traffic emissions contribution to PM_10_. The effects of those measures on PM_10_ mass concentrations in Munich were investigated with a semiparametric regression model for modeling PM_10_ levels adjusted for time, background pollution, public holidays and wind direction. The reduction of PM_10_ concentration after the introduction of the measures was larger at a traffic monitoring site (13.0 %, 19.6 % in summer, and 6.8 % in winter) and smaller in urban background (4.5 %, 5.7 % in summer, and 3.2 % in winter). The effect was most pronounced on Fridays and on the weekends in summer.

## 1. Introduction

In recent years, epidemiological studies have shown consistent associations between exposure to air pollution and cardio-respiratory morbidity and mortality [1,2,3]. The effects were partially attributed to traffic-related air pollutants such as fine particles, diesel soot or ultrafine particles in ambient air as well as other indicators of road traffic exposures, such as living or working close to major roads or nitrogen oxides [4,5,6,7]. Besides other ambient particle sources including e.g. industry and domestic heating, traffic emissions are supposed to have a significant impact on the local air quality and to contribute to the observed adverse health effects on humans. 

In 1999, the European Commission established limit values for PM_10_ and some other air pollutants in the Air Quality Daughter Directive 1999/30/EC [8], which was replaced in 2008 by the new Directive 2008/50/EC on ambient air quality and cleaner air for Europe [9]. The existing air quality guidelines for PM_10_ are currently being exceeded at many locations throughout Europe and Germany [10,11,12]. 

As traffic emissions are among the main contributors to anthropogenic pollutant emissions in the urban air [13], the reduction of traffic emissions offers an efficient strategy for reducing PM_10_ levels in urban air. One widely-used, non-technical measure to meet the policy targets for PM_10_ is the implementation of Low Emission Zones (LEZs). In Germany, a LEZ is a defined area (mostly located around the city centre) where the vehicles that enter have to meet certain emissions standards. For entering the LEZ all vehicles have to be identified by color coded windscreen badges which are directly linked to the corresponding stages of European emission standards (Euro 2: red; Euro 3: yellow; Euro 4: green). Petrol-driven vehicles equipped with a catalytic converter are principally assigned to the Euro 4 class and will be entitled to a green badge. In the first stage of operation all vehicles with a badge (red, yellow or green) are allowed to enter the LEZ. In stage 2, the LEZ can be accessed by vehicles displaying a yellow or green badge, whereas stage 3 of the LEZ allows access only to vehicles with a green badge. 

The first measure aiming reduction of PM_10_ concentrations in ambient air in Munich was the introduction of a law forbidding transit of vehicles heavier than 3.5 tons through the city area on 1 February 2008. This law forces all trucks without final destination in Munich to use the motorway ring A99 round around the city area. As a second measure the first stage of the LEZ became operative at 1 October 2008. In this stage, all vehicles with Euro 1 (or worse) were no longer allowed to enter and drive within the area of the LEZ. From 1 October 2010 the second stage of the LEZ in Munich became effective; all vehicles with Euro 2 (red sticker or without any sticker) are not allowed to enter the LEZ. Stage 3 of the Munich LEZ has been in operation since 1 October 2012 and only vehicles with green batches are allowed within the LEZ.

Currently, LEZs have been implemented in 13 European countries [14]. In Germany, 49 LEZs are in operation or in the planning stages [15]. Some local authorities in Germany estimated the expected impact of the LEZ on air quality prior to their implementation (mostly by dispersion modeling). The predicted reduction of PM_10_ mass concentration in ambient air, which is currently regulated and therefore the focus of our examination, ranges from 2% to 10 % depending on the characteristics of the specific LEZ, its valid stage and the number of exceptional permissions for cars that fail to meet the required emission standards. However, the verifying of the predicted reduction of PM_10_ levels by analyzing the measured PM_10_ mass concentrations is difficult, due to large influence of meteorological conditions on the PM_10_ levels [16].

Several studies about the benefit of traffic regulation measures in European cities were already published. PM_10_ and CO levels decreased after the introduction of the London congestion charging scheme [17,18] and PM_10_ and NO_X_ were reduced during a seven month test period of the congestion tax in Stockholm. Modest reductions in PM_10_ and PM_2.5_ were observed in four Danish cities as a consequence of the introduction of a LEZ [19]. Since the implementation of the London LEZ strong reductions of the particle number concentration were detected [20] and small changes in the PM concentration [21]. Boogaard *et al.* [22] found that local traffic policies were associated with reductions in PM_2.5_ in five Dutch cities. In spite of the challenges, there have already been a few papers analyzing the effects of the German LEZs on the improvement of air quality [16,23,24,25,26,27,28,29,30]. However, they mostly use rather simple (and varying) statistical approaches. Because these studies analyze different LEZs using different statistical methods, the results are difficult to compare insofar as different statistical approaches were used for the analyses. 

In the first study of LEZ impact in Munich, the adjustment for the influence of meteorological conditions on PM_10_ levels was conducted with a reference monitoring station located in a regional background area close to the city [25]. A relative reduction of PM_10_ levels has been seen at almost all involved monitoring sites and the relative decrease ranged from 5% up to 12 %. However, this analysis was applied on a rather short period and used descriptive statistical analyses only. Morfeld *et al.* [30] analysed the same data set using regression analyses for matched observations of subsequent years and have not found significant effects of the LEZ.

In our study, we extended the period under investigation and improved the statistical approach of the analysis. We restrict here our analysis only on the PM_10_ particle fraction. One reason for this restriction is that there are no data on other relevant particle characteristics (such as PM_2.5_, Black Smoke or particle number concentration) available for Munich. Secondly, LEZs were introduced in Germany as a primary measure for reducing PM_10_ and consequently the public debate about the effectiveness of LEZs is mainly focused on changes in this particle fraction. We compared the PM_10_ concentrations before and after the LEZ implementation by applying of a semiparametric statistical model with first-order autoregressive errors on data in a time resolution of one hour. The estimated PM_10_ levels were adjusted for PM_10_ exposure at the reference station, wind direction, season, time throughout a week, and public holidays. Two questions were of interest: First, we investigated, whether a significant overall reduction of PM_10_ levels adjusted for possible confounders could be detected following the implementation of the measures in Munich and whether the effect at the street site differed from the urban background site. Second, we examined the seasonal and diurnal variation of the air quality changes, which might have originated in the variations of the number of vehicles and of the composition of the vehicle fleet. 

## 2. Materials and Methods

### 2.1. Study Area and Selection of the Monitoring Stations

**Figure 1:**
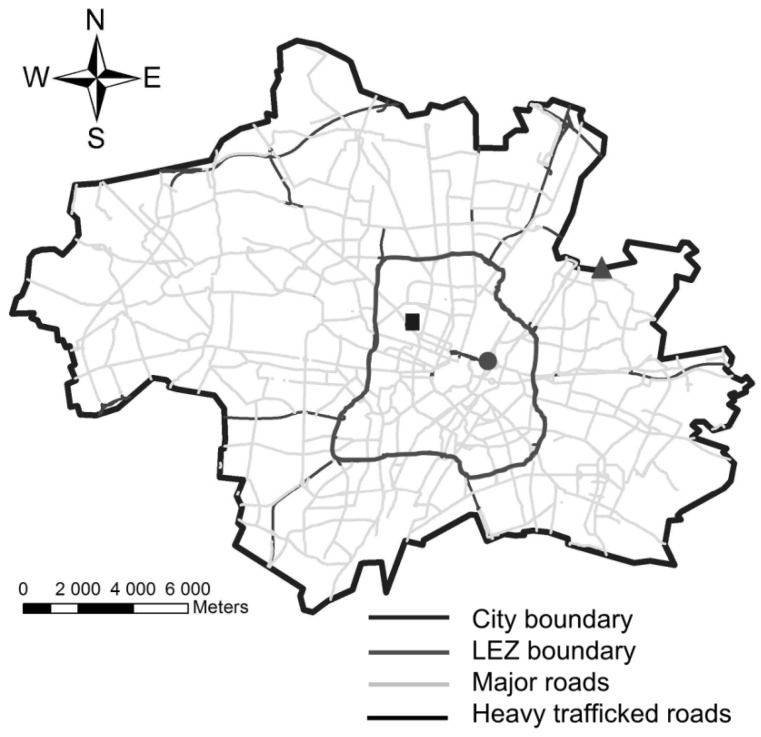
Locations of the LÜB monitoring sites in Munich, Germany: Prinzregentenstrasse (●), Lothstrasse (■), Johanneskirchen (▲).

The study was conducted in Munich, Germany. In 2011, Munich had a population of approximately 1.41 million inhabitants in an area of 310.4 km^2^ and there were approximately 700,000 registered cars [31]. The LEZ in Munich covers 44 km², which accounts for 14 % of the whole city area. However, 32 % of the city population lives in this area (Figure 1).

For our study, we obtained PM_10_ data collected at two monitoring sites located within the LEZ: one urban background site, Lothstrasse (measurement height: 4 m over ground) and one street site, Prinzregentenstrasse (measurement height: 2.9 m over ground; distance to road: 3 m; 39,000 vehicles/day in 2007–2010) [32,33]. The local traffic contributes with 22 % to the PM_10_ levels at the measurement site Prinzregentenstrasse and to 6 % at the site Lothstrasse [33]. A regional background site in the outskirts of the city and outside the LEZ in Johanneskirchen was selected as a reference site (measurement height: 4 m over ground; distance to road: 5 m). All three monitoring stations are operated by the Bavarian Environment Agency (Bayerisches Landesamt für Umwelt, Augsburg, Germany) as part of the Bavarian Monitoring System for Air Quality (LÜB: Lufthygienisches Landesüberwachungssystem Bayern) (Figure 1). The remaining three monitoring stations were not involved in the analysis and are not shown in Figure 1. Two of them (Luise-Kiesselbach-Platz, Landshuter Allee) are located at the border of the LEZ and one (Stachus) were excluded as construction and tram track maintenance was conducted between August 2008 and April 2010 in the vicinity of this monitoring site.

### 2.2. Study Period and Data

In our analysis, we compared the PM_10_ concentrations measured prior to the implementation of any air quality measures (period 1) with the PM_10_ levels measured after the measures became effective (period 2). 

Data on PM_10_ mass concentrations in an hourly time resolution were used for the analysis. The analysis period 1 was selected from 1 February 2006 until 31 January 2008. In the period between 1 February 2008 and 30 September 2008, only a truck transit ban was effective. This period was excluded from the analysis because it was too short to draw general conclusions. The examination period 2 (both transit ban for heavy-duty vehicles and LEZ in stage 1 were effective) covered the rest of the data between 1 October 2008 and 30 September 2010. PM_10_ records at the Prinzregentenstrasse were only available until 31 June 2010, because the station was closed. The PM_10_ values on 1 January were excluded from the analysis for each year due to the traditional New Year’s Eve fireworks.

Hourly measurements of the wind direction in 36 angle categories representative for the city of Munich (measured ca. 4 km northwest of the inner city of Munich) were obtained from the German National Meteorological Service. 

### 2.3. Statistical Analysis

The urban PM_10_ levels were assumed to be mainly driven by the following factors: The measurements at the reference station represented the regional background pollution level, which was mostly not affected by the measures. Therefore, the PM_10_ levels of the reference station reflected the changes of the PM_10_ levels of the station of interest owing to meteorological conditions, the background PM_10_ levels and long term temporal trends in PM_10_. Despite large scale effects of the wind direction, which were represented through the reference station, local effects of the wind direction occur due to the position of the measurement stations in the local vicinity, e.g. regarding to nearby factories or park areas.

Since our data comprise only four years of measurements, long term temporal trends at the stations within the LEZ beyond the trends at the reference station could not be considered in the model and are of minor relevance. However, seasonal variation in PM_10_ concentration occurred (see Supplemental Material, Figure S1), which possibly may change the effect of the measures. Hourly PM_10_ levels are subject to daily cyclic variation due to heating and also due to traffic; therefore, the measures may also affect the temporal pattern of PM_10_ levels. Public holidays during weekdays may yield deviations from the usual concentration levels.

A semiparametric model with first-order autoregressive errors (refer to Clifford *et al.* [34] for a Bayesian implementation of a similar model for ultrafine particle number concentrations) was used to estimate the association between air pollution and the introduction of measures:


log(*PM*_10*x*_) = β_0_ + β_1_ log(*PM_10ref_*) + β_SM_ · I_SM_ + β_W_ · I_W_ + β_WM_ · I_WM_ + *f*_S_(*hour*) · I_S_ + *f*_SM_(*hour*) · I_SM_ + *f*_W_(*hour*) · I_W_ + *f*_WM_(*hour*) · I_WM_ + *f*_wd_(*wind direction*) + β_2_(*public holiday*) + ε . (1)

The effects of the corresponding covariates were denoted with β.The outcome variable was the logarithmically transformed PM_10_ mass concentration at an urban station, which is denoted by log(*PM_10_*_x_). I_S_, I_W,_ I_SM_, I_WM_ denote the indicator function for “summer without measures”, “winter without measures”, “summer with measures” and ”winter with measures”, respectively. The winter season was defined from October to March, the summer season from April to September.

The following variables were considered as confounding factors: logarithmically transformed PM_10_ levels at the reference station (log(*PM_10_*_ref_)), a smooth, cyclic effect based on P-splines with maximum four degrees of freedom of the wind direction, *f*_wd_(*wind direction*), and an indicator for public holidays (*public holiday*). Adjusting for the PM_10_ measurements at the reference station prevents from “regression to the mean” [35], improves the power of the model in comparison to the analysis of differences [36] and allows flexible and simple adjustments for other confounders. We selected the confounder variables by a priori considerations. Since we include an adjustment by the reference station with similar temperature and precipitation, we did not use these variables in the confounder model. Since wind direction has a local effect, which differs from the effect at the reference station, we included the variable *wind direction* in our model.

In addition, the model was adjusted for deterministic seasonal components, similar to [37]. The effect of the measures (M) was analyzed separately for summer (S) and winter (W) to allow for seasonal variability. Daily and daytime-specific deviations were modeled with an hourly-resolved weekly season-specific trend, *f*_S_(*hour*) · I_S_, *f*_SM_(*hour*) · I_SM_, *f*_W_(*hour*) · I_W_, *f*_WM_(*hour*) · I_WM_ Cyclic penalized splines were used as basis functions for a smooth nonparametric estimation. Permitting a maximum number of 49 degrees of freedom, the model reached enough flexibility to describe the daytime dependent variability of the traffic.

Percentage changes of PM_10_ levels were modeled through logarithmic concentration levels [37]. In particular, it was suggested that the measures, the public holidays and the seasons yielded to percentage effects on the PM_10_ concentration.

Note, that the usage of the semiparametric model lessens the problem of scale. Furthermore, the highly skewed distribution of the PM_10_ mass concentration was another reason for using the logarithmic transformation.

Since the measurements are not independent, an autoregressive process of order 1 is simultaneously modeled for the error term *ε*.

The overall effect of the measures was examined for each of the two seasons with a test on the hypotheses whether the effect coefficients for winter and summer differ between the periods with and without measures:

*H*_0_ : β_W_ = β_WM_,β_SM_ = 0 (2)

Day-specific effects were investigated using an appropriate linear combination of the effect coefficients, representing the area between the smooth effect with and without measures. The inference for the day-specific effects was based on the asymptotic normality of linear combinations (for more details see Supplemental Material, “Modelling of day specific effects”).

Statistical calculations were conducted using R [38], version 2.15.3; semiparametric models were estimated with the package “mgcv” [39], version 1.7-22.

## 3. Results

### 3.1. Data Description

**Figure 2:**
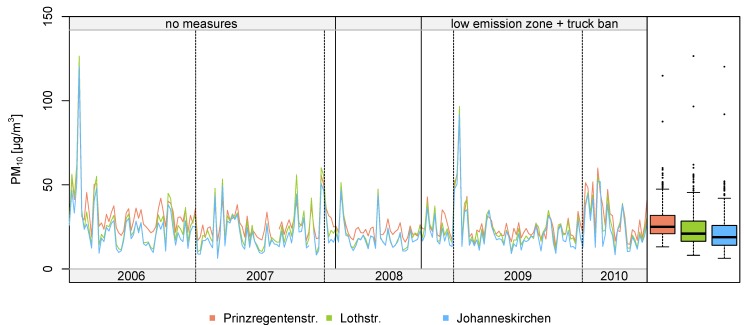
Time series of weekly averages of PM_10_ concentrations at three monitoring sites in Munich, Germany (Prinzregentenstrasse, Lothstrasse, and Johanneskirchen). The three boxplots (on the right-hand side) describe the respective distributions the PM_10_ concentrations at the three sites ignoring the temporal structure.

Figure 2 shows the weekly means of PM_10_ concentrations at the two monitoring sites within the LEZ (Prinzregentenstrasse and Lothstrasse) and at the reference site in Johanneskirchen. Weekly means were chosen to display the trend of PM_10_ levels for reasons of clarity and reducing the point-to-point variation.

The PM_10_ concentrations were higher in the winter season and lower in the summer season. In particular, elevated PM_10_ levels were observed between January and March. The means of the unadjusted PM_10_ mass concentrations before and after the implementation of measures are given in Table 1.

At both urban stations, a decrease of PM_10_ in period 2 (compared to period 1) was observed. The decrease at Prinzregentenstrasse (−14.0% in summer, −1.9% in winter) was larger in summer and similar in winter as at Lothstrasse (−2.3% in summer, −2.5% in winter). At the reference station in Johanneskirchen, also differences between the two periods were detected (−2.1% in summer, −0.8% in winter). The PM_10_ concentration was on average 27.6% at Prinzregentenstrasse and 12.5% at Lothstrasse higher (median percentage difference) than at the reference station. While considering mere mean concentrations the impact of confounding factors like meteorological conditions were disregarded; the regression analysis in section 3.2 allowed for these confounders. The Spearman correlation coefficients between the hourly means of PM_10_ concentration measured at the three monitoring sites were high and ranged from 0.69 to 0.88, the correlations between the daily averages were even slightly stronger (ranged from 0.83 to 0.96). The hourly PM_10_ concentrations at the three monitoring sites were highly autocorrelated with autocorrelation coefficients between 0.87 and 0.92. The temporal trend of the annual means observed at the urban background site Lothstrasse and the regional background site (as the reference site) in Johanneskirchen was very similar. It indicates that both sites were not influenced by any local sources of PM. Even more, the PM_10_ values obtained at the measurement site in Johanneskirchen were very similar and strong correlated with the PM_10_ values observed at a regional background site located at the campus of the Bavarian Agency for Environmental Protection in Augsburg, Germany (about 90 km linear distance from Johanneskirchen). 

**Table 1:** PM_10_ means in Munich for the periods with (October 2008–September 2010) and without (February 2006–January 2008) measures and the corresponding differences in % separated by season (Summer: April–September; Winter: October–March).

Measurement Station	Season	Without Measures		With Measures		Percentage Difference
		*n*	PM_10_ mean (SD)	*n*	PM_10_ mean (SD)	
Prinzregentenstr.	Summer	8,200	27.2 (14.3)	6,535	23.4 (14.5)	−14.0
	Winter	8,562	30.8 (21.6)	8,676	30.2 (23.6)	−1.9
Lothstr.	Summer	8,769	21.3 (12.9)	8,730	20.8 (15.3)	−2.3
	Winter	8,520	28.3 (23.6)	8,687	27.6 (22.0)	−2.5
Johanneskirchen	Summer	8,765	19.3 (12.2)	8,768	18.9 (12.3)	−2.1
	Winter	8,451	24.3 (21.6)	8,686	24.5 (20.8)	0.8

### 3.2. Statistical Modeling

According to equation (1), the PM_10_ concentrations at a specific monitoring site were calculated for “summer without measures” and “winter without measures” as well as “summer with measures” and “winter with measures”, respectively. In Table 2, the relative differences between the periods with and without measures are shown for Prinzregentenstrasse (street site) and Lothstrasse (urban background site), and indicated separately for the summer and winter season.

**Table 2:** Change of PM_10_ concentration ^a^ in period 2 when compared to period 1 at Prinzregentenstrasse and Lothstrasse.

Measurement Station	Summer			Winter		
	effect	confidence interval	*p*-value	effect	confidence interval	*p*-value
Prinzregentenstr.	−19.63%	(−22.75%, −16.52%)	<0.001	−6.80%	(−10.14 %, −3.47 %)	<0.001
Lothstr.	−5.73%	(−7.71%, −3.74%)	<0.001	−3.18%	(−5.24 %, −1.11 %)	0.003

Note: adjusted for exposure at the reference station, wind direction, day of the week, time of the day and public holidays.

The effects of the air quality measures differ at the two measurement stations as well as between the seasons. A stronger reduction in PM_10_ mass concentrations was observed at the street site; on average 19.6% (5.4 µg/m^3^, *p*-value: <0.001) in summer and 6.8% (2.1 µg/m^3^, *p*-value: <0.001) in winter, respectively. At the urban background site Lothstrasse smaller decreases were estimated: 5.73% (1.1 µg/m^3^, *p*-value: <0.001) in summer and 3.18% (0.7 µg/m^3^, *p*-value: <0.003) in winter. If we had not have determined separate effects for the two seasons, the reduction of the PM_10_ concentration by the measures at Prinzregentenstrasse would have been estimated with 13.0% (*p*-value: <0.001) and with 4.5%; (*p*-value: <0.001) at Lothstrasse.

**Figure 3:**
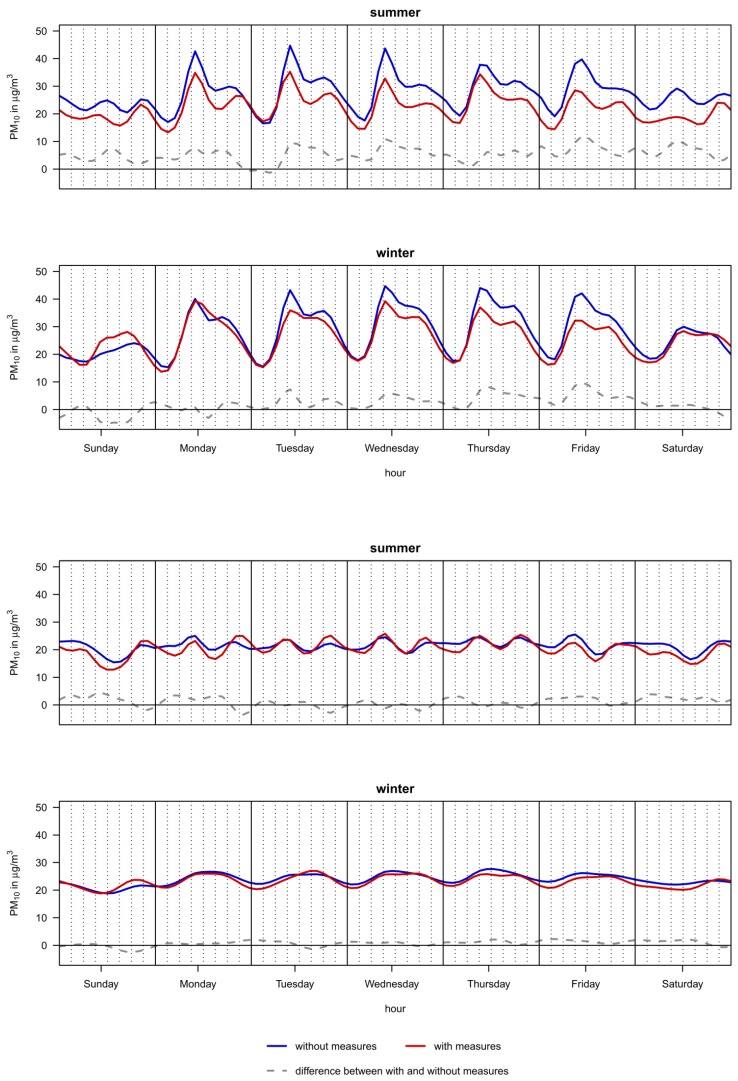
Modeled hourly concentrations of PM_10_ at Prinzregentenstrasse (first and second chart) and Lothstrasse (third, fourth chart) adjusted for PM_10_ at the reference station, wind direction and public holidays.

Figure 3 shows the temporal patterns of the modeled PM_10_ concentrations at Prinzregentenstrasse and Lothstrasse for the periods with and without measures (adjusted for PM_10_ concentration at the reference station, wind direction and public holidays). Temporal variability of PM_10_ levels occurred between the seasons, the weekdays and times of day. The concentrations were higher in the winter months. The morning and afternoon rush hour peaks during the working days were clearly visible especially at Prinzregentenstrasse. The morning peak in the summer months was more clearly separated from the afternoon peak as in the winter season. The efficiency of the measures depended on the time of the day (see also Supplemental Material, Figure S2) and followed a diurnal pattern. Due to the implemented measures the PM_10_ burden was stronger reduced during hours with higher relative and absolute PM_10_ mass concentration, *i.e.* between the morning and afternoon rush hour peaks. The rush hour peaks themselves were reduced and there seemed to be lesser spillover from the morning to the afternoon. Furthermore, the improving of air quality during the nights on workdays was faster at the street site. The effect of the measures vanishes during night-time of the first days of the working week.

**Figure 4:**
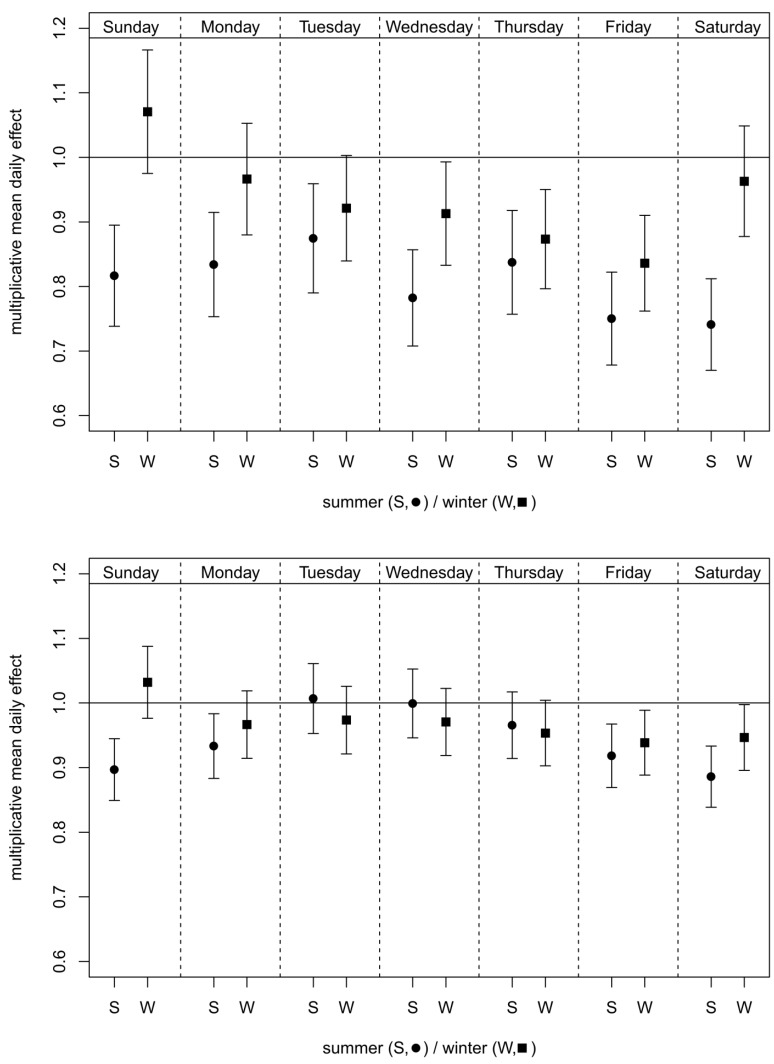
Mean daily effects of the measures stratified by season and day of the week with 95% confidence intervals for Prinzregentenstrasse (**above**) and Lothstrasse (**below**).

The mean daily effects of both measures (stratified by season and week day) are shown in Figure 4 for each day of the week separately. In the summer season, the effect of the measures for each day of the week was at the street site stronger than in winter, whereas this tendency is not observed at the background site. However, at both sites the strongest effects were observed on Fridays (−25.0% in summer and −16.4% in winter at street site, −8.2% in summer and −6.1% in winter at the background site) and Saturdays (−25.9% in summer at street site, −11.4% in summer and −5.3% in winter at the background site). On Sunday a strong seasonal dependency of the effect was observed: the measures were only effective in summer.

The effects of the linearly modeled confounding covariates are displayed in Supplemental Material, Table S1. The logarithmic values of the reference station had a significant, additive effect of log(1.381) = 0.323 at Prinzregentenstrasse and log(1.972) = 0.679 at Lothstrasse. Public holidays led to a reduction of PM_10_ levels in the ambient air by approximately 13%. Since the estimated autocorrelation coefficient was relatively high (Prinzregentenstrasse: ρ = 0.70, Lothstrasse: ρ = 0.54), the bigger part of the autocorrelation of the PM_10_ measurements could not be explained through the inclusion of the smooth components in the predictor. The model for the measurements at Prinzregentenstrasse explained 74.5% of the variability in the data and the model for the Lothstrasse 83.0%.

The shape of the smooth effect of wind direction indicates decreased PM_10_ levels, if the wind blew from the South or West at Lothstrasse and if the wind blew from North or East at Prinzregentenstrasse (data not shown). We focused our analysis on PM_10_, because the reason for the establishment of the LEZ exceeded PM_10_ limit values.

## 4. Discussion

### 4.1. Impact of the Measures on PM_10_ Mass Concentration

We investigated whether changes in PM_10_ levels after the introduction of a truck transit ban through the city area and the implementation of the first stage of the LEZ in Munich could be detected by analysis of emission data on PM_10_ mass concentration collected at an urban background and a street monitoring site. The comparison of the PM_10_ mass concentrations (adjusted for exposure at the reference station, wind direction, day of the week, time of the day and public holidays and calculated separately for summer and winter seasons in a semiparametric model with first-order autoregressive errors) showed a large relative decrease of PM_10_ levels at the street site (13.0%, *p*-value: <0.001), whereas the relative decrease observed at the urban background monitoring site was smaller (4.5%, *p*-value: <0.001). The decrease of PM_10_ mass concentration predicted in Munich by dispersion modeling ranges between 2% and 10 % depending on the monitoring sites and the active stage of the LEZ [40]. The maximal reduction (up to 10 %) was predicted only for the third stage of the LEZ. The changes of PM_10_ concentrations detected at the street site in our study are larger than the reductions predicted a priori and are also larger than those observed in the most other German cities [16,23,24,26,27,28,29]. 

In general, the implementation of LEZ could influence the composition of the car fleet as well as the traffic intensity. The percentage of registered vehicles without any badge (Euro 1 or less) decreased during the time period 2007–2010 from 9.2% to 2.5% for passenger cars and from 31.5% to 24.1% for trucks, respectively. In the same time the percentage of vehicles with green badge increased from 78.4% to 89.6% for passenger cars and from 19.0% to 36.1% for trucks, respectively. Those changes are especially pronounced between the years 2007 and 2008, it means immediately before the implementation of the LEZ in Munich [41]. Such an extraordinary modernization of vehicle fleet in the city towards low-emission cars was reported also for Berlin [28]. Note that there is only information about the in Munich registered vehicles; no such information is available about the car fleet composition in flowing traffic in the city. Regarding the flowing traffic it can be assumed that the older vehicles are less often in use compared to the newer vehicles. 

For the dispersion modelling, it was assumed that the traffic intensity remained constant over the time period 2007–2010. However, the analyses presented here are not only considering the impact of the LEZ alone, but also the additional impact of the transit ban for all trucks. The transit ban for trucks could affect the PM_10_ levels even to a larger extent than the LEZ, which operated in the analyzed period in the first stage only. It leads not solely to a reduction of particles emitted by vehicle exhaust, but also to a reduction of particles originated from tyre and brake wear or dust re-suspension. Due to the ban on driving for trucks on Sunday, the effect for Sunday can be directly ascribed to the implementation of the LEZ. A similar pattern was found for Saturday, but only at Prinzregentenstrasse. This lead us to the assumption that in winter, the vehicle fleet on weekends in the city and on Sundays in the urban background was the same before and after the introduction of the LEZ, whereas this was not the case during the summer season.

In the previous study estimating the LEZ impact in Munich, a slightly weaker effect of 12.3% relative reduction of PM_10_ mass concentration at Prinzregentenstrasse was found [25]. The analysis was based on the comparison of relative PM_10_ concentration changes by a reference station. However, such analysis of the quotient between the specific monitoring station and the reference station neglects the uncertainty of the measurements at the reference station. Further regression analyses on the ratio as used in the previous study revealed a comparably poor model fit (data not shown). This is also denoted by the strong deviation of the estimation of the intercept from 1 in our analysis. For comparison, we also analyzed the same period as described in Cyrys *et al.* [25] by use of the model applied in our study. We only found negligible (statistically insignificant) changes of the PM_10_ mass concentration (Prinzregenstrasse −1.05%, *p*-value: 0.855; Lothstrasse: 2.42%, *p*-value: 0.499), as it was similarly found by Morfeld *et al.* [30]. For further comparisons of the approach used in our study with other modelling approaches we refer to the “comparison of different modeling approaches” section in the Supplemental Material.

The results of our study are not directly comparable to the results obtained for other measures of traffic reduction, which were already introduced in some European cities. We analyzed here the common effect of the implementation of LEZ and transit ban for trucks in Munich and we are aware that such combination is not that common. Even if we might be able to evaluate the effects of the LEZ and transit ban for trucks separately, the comparison with other cites might remain difficult, as the regulations and areas of LEZ’s differ from city to city. The following discussion should compare rather roughly the range of the effects observed for different measures across Europe. Several studies analyzed the impact of congestion charging in London [17,18,42]. Atkinson *et al.* [17] observed reductions in PM_10_ only at the background monitor. The study conducted by Beevers and Carslaw [18] indicated that NO_x_ and PM_10_ emissions have been reduced by about 12% in the charging zone, whereas the study of Tonne *et al.* [42] showed that the congestion charge schema led to only modest reductions in air pollutant concentrations across Greater London, but greater reductions in the charging zone. Ellison *et al.* [21] found that the LEZ in London had a significant effect on the composition of the vehicle fleet in London and reduced the PM_10_ concentrations.

Johansson and colleagues [43] assessed the effect of traffic congestion in Stockholm and concluded that the annual average NO_x_ and PM_10_ concentrations along the most densely trafficked streets would be lower by up to 12% and 7%, respectively. Note that the effects in the studies of Beevers and Carslaw [18], Tonne *et al.* [42] and Johansson *et al.* [43] were analyzed by dispersion modelling combined with regression calculation and were not verified by air quality measurements. PM_2.5_ concentrations in Copenhagen were reduced by 5% after the introduction of the LEZ [19]. Unadjusted mean pollutants concentrations were lower after the implementation of the LEZ in five Dutch cities; the reduction in PM_2.5_ levels was larger at urban streets (31%) than in the suburban background (20%) [22].

The public debate is often focused solely on PM_10_ concentrations (as this parameter is currently regulated) without taking into account that only the toxic fraction of PM_10_ causes adverse human health effects [2,3,16]. Due to combustion processes particles originating from traffic exhibit a higher toxicity than particles from other sources; especially diesel-engine vehicles, which produce about 12% of the mean PM_10_ exposure of the German population [44], emit these more toxic particles.

Hence, the effectiveness of LEZ could be analyzed more precisely if Black Smoke (as marker for diesel soot) or the organic fraction of particles would be measured in ambient air instead of total PM_10_ concentration [16]. Unfortunately, in Germany no routine measurements of Black Smoke concentrations in ambient air are conducted. Quadir and colleagues [45] reported recently significant lower concentrations for elemental carbon and some of particulate organic compounds after the introduction of the LEZ in Munich (the data were collected during special monitoring campaigns and not routinely by the monitoring network). Source apportionment analysis showed a reduction of traffic factor contribution by 60% after the implementation of the LEZ [45]. Also in Berlin the concentration of soot particles decreased 2010 by 52% compared with 2007 [29]. As climatic conditions in the years 2008–2010 were adverse when compared to 2007, the authors attribute these results to the reduced traffic soot emissions. Currently, a debate about the pollution through PM_2.5_ emerges, but the data material is insufficient because the monitoring of PM_2.5_ is only at its early stages.

In addition to dispersion modelling, estimating the expected changes of PM_10_ mass concentration in the ambient air, our analysis evaluates the effects of the measures by analysis of the measured PM_10_ values. However, the limitation of this strategy is, that also long-term changes of PM_10_, which could not be explained by the included predictors, especially by the PM_10_ levels of the reference station (for example changes in heating habits), are completely attributed to the LEZ effect.

### 4.2. Seasonal and Diurnal Variation of the Changes in PM_10_ Levels

The second objective of this study was the examination of the seasonal and diurnal variation of the detected air quality improvements. The extent of the PM_10_ reduction at the background sites was largely similar in both seasons. In the urban background, exhaust particles represent a smaller fraction of fine particles compared to street site and the composition of particles varies less between winter and summer. On the contrary the difference between the two seasons was more pronounced at the street site, where the overall reduction of PM_10_ mass concentrations was considerable larger as at the background site. At this site, the reduction of PM_10_ levels due to the measures was larger during the summer season and smaller during the winter season. In winter, additional particle sources (such as domestic heating, wood combustion or combustion of other fossil fuels) contribute significantly to the PM_10_ mass concentrations in the ambient air. Also the contribution of re-suspended dust to fine particles concentration in the ambient air increases in winter due to the application of road salt for deicing. In addition, the generation of secondary aerosols such as nitrate or sulfate is more intensive in winter. Consequently, exhaust particles represent a smaller fraction of the fine particles in winter than in summer. Therefore, the measures regulating only the exhaust particles became less effective in the winter period. In addition, adverse meteorological conditions leading to increased PM_10_ levels from local mobile as well as stationary sources are occurring more frequently in the winter season. Our analysis suggests that in such episodes the influence of the implemented measures regulating the car exhaust is limited and that air quality is dominated by other unaffected mobile and stationary sources.

The reduced rush hour peaks may indicate that a larger proportion of old cars no longer accessed the city during midday. Alternatively, the contribution of aged particles from the morning rush hours during day and night time might be shifted due to the reduction in diesel particles. Further, the temporal varying effect of the measures could be caused by the same reason as the differences between winter and summer season. One has to keep in mind that the morning and afternoon rush hour peaks seem to be more separated in summer not due to differences in traffic flow between summer and winter, but as a result of the combination of increased traffic intensity and increased solar radiation in summer.

## 5. Conclusions

In our study we evaluated the effectiveness of two measures (a truck transit ban through the city area and implementation of LEZ) on the reduction of PM_10_ mass concentrations in the ambient air in Munich, Germany. The analysis of the routinely collected PM_10_ mass concentrations data by a semiparametric regression model showed statistically significant reduction of PM_10_ levels at a monitoring site located in the direct vicinity of a highly frequented road and to a lesser extent at an monitoring site located in the urban background. The statistical regression modeling was essential to identify the size of the effect. The magnitude of the effect at the street site was larger in summer season; smaller seasonal variation was observed at the urban background site. In general, the magnitude of the effect depends on day of the week, time of the day and location of the monitoring site.

Our analysis indicates that the assessment of the impact of measures aiming improvement of air quality in urban air could be conducted by use of routinely collected PM_10_ data. However, as the expected reduction of PM_10_ concentration after implementation of LEZ is in order of about 10%, the evaluation of this measure by PM_10_ data remains difficult; other particulate variables, such as PM_2.5_, Black Smoke, or particulate organic compounds are recommended for such evaluation.

## Supplementary Files

Supplementary File 1 Supplementary Information (PDF, 397 KB)
