# Supplementary material for: Evaluation of the Impact of Low Emission Zone and Heavy Traffic Ban in Munich (Germany) on the Reduction of PM10 in Ambient Air
Source: Int J Environ Res Public Health. 2014 May 13;11(5):5094–112. doi: 10.3390/ijerph110505094 (PMC4053866; doi:10.3390/ijerph110505094)
Supplement: Supplementary File 1 — Supplementary Information (PDF, 397 KB) [file ijerph-11-05094-s001.pdf]

Supplementary Information

Evaluation of the Impact of Low Emission Zone and Heavy Traffic Ban in Munich (Germany) on the Reduction of PM_{10} in Ambient Air

Figure S1. Seasonal variation of PM10 concentration at Prinzregentenstrasse.

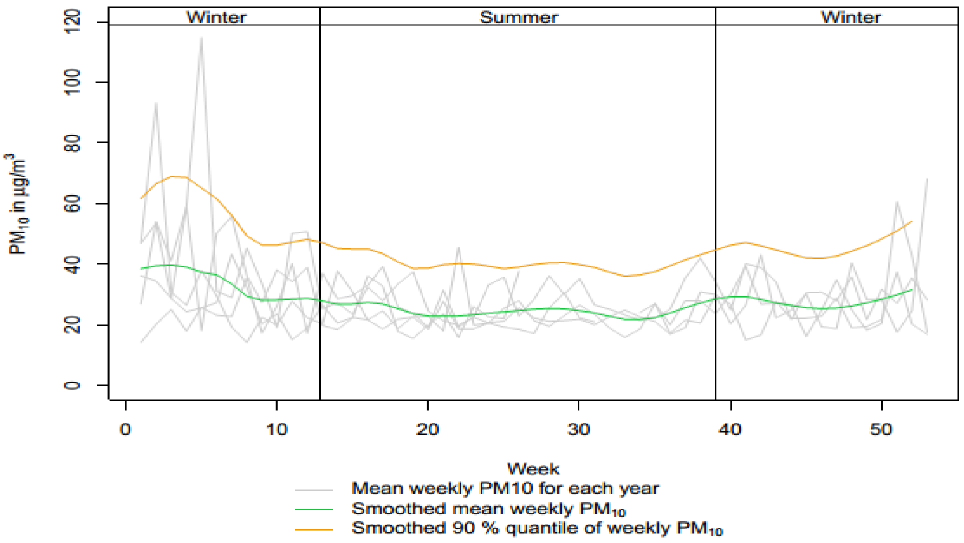

Figure S2. Mean temporal multiplicative effect of the measures on weekdays.

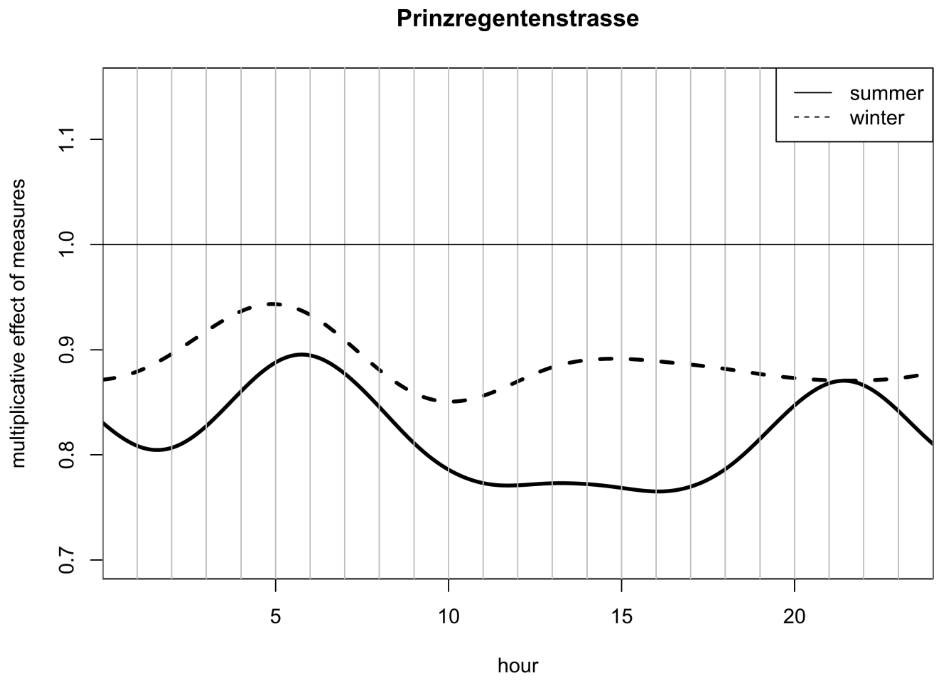

Figure S2. *Cont.*

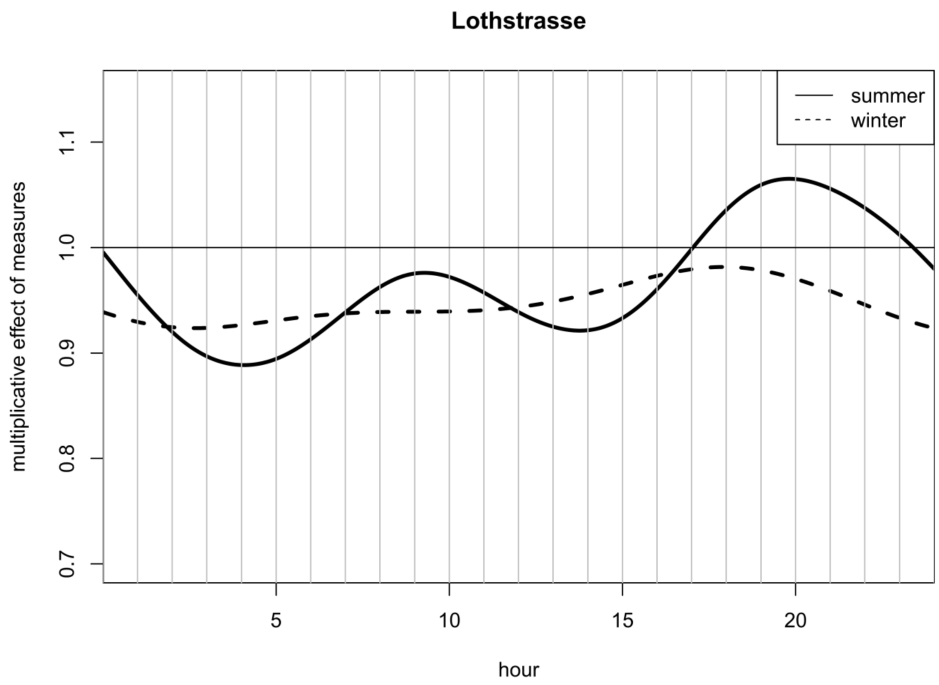

Table S1. Multiplicative effects of the linearly modelled confounding covariates at Prinzregentenstrasse and Lothstrasse.

**Prinzregentenstrasse**	**mult. effect**	**Confidence Limits**		***p*-value**
		**lower boundary**	**upper boundary**	
Intercept	9.759	9.090	10.428	<0.001
log(PM_{10}) reference station	1.381	1.366	1.397	<0.001
public holidays	0.868	0.817	0.919	<0.001
**Lothstrasse**	**mult. effect**	**Confidence Limits**		***p*-value**
		**lower boundary**	**upper boundary**	
Intercept	2.708	2.582	2.834	<0.001
log(PM_{10}) reference station	1.972	1.956	1.988	<0.001
public holidays	0.874	0.839	0.909	<0.001

Modelling of Day-specific Effects

Day-specific effects are examined using the area between the smooth effect with and without measures. For this purpose, a linear combination $\boldsymbol{C\beta}$ of the effect coefficients $\boldsymbol{\beta}$ and an adequate matrix $\boldsymbol{C}$ containing linear constraints was built for each season.

The construction of the constraint matrix $\boldsymbol{C}$ is explained in the following. The hypothesis for testing is, that the areas between the smooth effect curves $f_{\mathrm{S}}(hour)$ and $\beta_{\mathrm{SM}} + f_{\mathrm{SM}}(hour)$ and $\beta_{\mathrm{W}} + f_{\mathrm{W}}(hour)$ and $\beta_{\mathrm{WM}} + f_{\mathrm{WM}}(hour)$, respectively, are not equal to zero for a certain day. We will

show in the following, that this hypothesis can be written as a linear hypothesis on the parameter vector $\boldsymbol{\beta} = (\beta_0, \beta_1, \beta_2, \beta_{\mathrm{SM}}, \beta_{\mathrm{W}}, \beta_{\mathrm{WM}}, \boldsymbol{\beta}_{f_{\mathrm{S}}}, \boldsymbol{\beta}_{f_{\mathrm{SM}}}, \boldsymbol{\beta}_{f_{\mathrm{W}}}, \boldsymbol{\beta}_{f_{\mathrm{WM}}}, \boldsymbol{\beta}_{f_{\mathrm{wd}}})^{\mathrm{T}}$. Let $A_{\mathrm{d}}^{\mathrm{s}}$ denote the area under the smooth effect curve for season *s*, $s \in \{\mathrm{S,W}\}$, without measures and day *d* and $A_{\mathrm{d}}^{\mathrm{sM}}$ the area for the same season and day but with measures.

The statistical null hypotheses can be formalized as $\mathrm{H}_0\colon A_{\mathrm{d}}^{\mathrm{sM}} - A_{\mathrm{d}}^{\mathrm{s}} = 0$, with $\mathrm{s} \in \{\mathrm{S}, \mathrm{W}\}$. Some transformations for the left side yield with *K* as the number of degrees of freedom of the smooth hour effect and *B* as the P-spline basis functions:

$$\begin{aligned} A_{\mathrm{d}}^{\mathrm{sM}} - A_{\mathrm{d}}^{\mathrm{s}} &= \frac{1}{7}A^{\mathrm{sM}} + \int\limits_{hour \in \mathrm{d}} f^{\mathrm{sM}}(hour)\mathrm{d}hour - \frac{1}{7}A^{\mathrm{s}} - \int\limits_{hour \in \mathrm{d}} f^{\mathrm{s}}(hour)\mathrm{d}hour \\ &= \frac{1}{7}A^{\mathrm{sM}} + \int\limits_{hour \in \mathrm{d}} B(hour)^{\mathrm{T}}\boldsymbol{\beta}_{\mathrm{f_{sM}}}\mathrm{d}hour - \frac{1}{7}A^{\mathrm{s}} - \int\limits_{hour \in \mathrm{d}} B(hour)^{\mathrm{T}}\boldsymbol{\beta}_{\mathrm{f_s}}\mathrm{d}hour \\ &= \frac{1}{7}A^{\mathrm{sM}} + \sum_{k=1}^{K-1}\int\limits_{hour \in \mathrm{d}} B(hour)^{\mathrm{T}}\beta_{\mathrm{f_{sM}}}^{k}\mathrm{d}hour - \frac{1}{7}A^{\mathrm{s}} - \sum_{k=1}^{K-1}\int\limits_{hour \in \mathrm{d}} B(hour)^{\mathrm{T}}\beta_{\mathrm{f_s}}^{k} hour \\ &= 24\beta^{\mathrm{sM}} + \sum_{k=1}^{K-1}\beta_{\mathrm{f_{sM}}}^{k}\int\limits_{hour \in \mathrm{d}} B(hour)^{\mathrm{T}}\mathrm{d}hour - 24\beta^{\mathrm{s}} \\ &\quad - \sum_{k=1}^{K-1}\beta_{\mathrm{f_s}}^{k}\int\limits_{hour \in \mathrm{d}} B(hour)^{\mathrm{T}}\mathrm{d}hour \end{aligned} \tag{1}$$

With

$$\boldsymbol{C}^{\mathrm{S}} = \left(0, 0, 0, 24, 0, 0, , -\int\limits_{hour \in \mathrm{d}} B(hour)^{\mathrm{T}}\mathrm{d}hour, \int\limits_{hour \in \mathrm{d}} B(hour)^{\mathrm{T}}\mathrm{d}hour, 0, \dots, 0\right) \tag{2}$$

and

$$\boldsymbol{C}^{\mathrm{W}} = \left(0, 0, 0, 0, -24, 24, 0, \dots, 0, -\int\limits_{hour \in \mathrm{d}} B(hour)^{\mathrm{T}}\mathrm{d}hour, \int\limits_{hour \in \mathrm{d}} B(hour)^{\mathrm{T}}\mathrm{d}hour, 0, \dots, 0\right) \tag{3}$$

the hyotheses can be written as $\mathrm{H}_0\colon \boldsymbol{C}^{\mathrm{s}}\boldsymbol{\beta} = 0$, with $\mathrm{s} \in \{\mathrm{S}, \mathrm{W}\}$. The test statistics T^S and T^W are given by $T^S = \frac{\boldsymbol{C}^{\mathrm{S}}\hat{\boldsymbol{\beta}}}{\boldsymbol{C}^{\mathrm{S}}\hat{\boldsymbol{\beta}}\boldsymbol{C}^{\mathrm{S}^{\mathrm{T}}}}$ and $T^W = \frac{\boldsymbol{C}^{\mathrm{W}}\hat{\boldsymbol{\beta}}}{\boldsymbol{C}^{\mathrm{W}}\hat{\boldsymbol{\beta}}\boldsymbol{C}^{\mathrm{W}^{\mathrm{T}}}}$, which are asymptotically normal distributed.

Comparison of Different Modelling Approaches

Model choice may greatly influence the inference on the effectiveness of air quality measures. Alternative modelling strategies including the model type, the type of the effect and the predictor set are discussed in the following.

Morfeld *et al.* [1] proposed a regression analysis design on the concentration differences between the year after the introduction of measures and the previous year. The analysis of differences complicates the adjustment for confounding variables, particularly the wind direction and categorical factors like an indicator for public holidays. Allowing for the measurements before the introductions of

the LEZ from the reference station introduces collinearity with the baseline values of the focused station, because the measurements of both stations are highly correlated. Moreover, the power of the model will be insufficient, because the variability of the effect will be overestimated since the concentration differences were modeled as outcome [2]. The planned analysis will involve data from several German cities. Indeed, the effect of the measures is allowed to vary between the different cities, but the confounding factors are postulated to have equal effects in each city. Especially considering the reference station, differing effects should be assumed.

The model suggested by Morfeld *et al.* [1] assumes an additive impact of the predictors on the PM_{10} mass concentration. Using additive effects (eq. 1 without taking the logarithm of the PM_{10} concentrations) in our model, we estimated a reduction of 3.90 μg/m^3 (13.84%, *p*-value: <0.001) in summer and 0.50 μg/m^3 (1.71%, *p*-value = 0.372) in winter at Prinzregentenstrasse (Lothstrasse: 0.33 μg/m^3, 1.34%, *p*-value = 0.285 in summer and 0.75 μg/m^3, 2.71%, *p*-value = 0.016 in winter). The effects are lower than our results obtained by using logarithmic PM_{10} concentrations in the model. However, an additive structure does not fit the assumption of conditional normality of the highly-skewed response variable. Moreover, we presume a percentage change of the vehicle fleet and thus, of the PM_{10} mass concentration.

Involving the measurements of the reference station allows for the complex, largely unknown effect of the weather conditions. Sensitivity analyses for the model of the Prinzregentenstrasse station showed only a slight amelioration of the model fit and only minor changes of the measures' effect, if weather conditions (precipitation, temperature, relative humidity, wind speed) were also considered in the model.

Moreover, traffic intensity is a major predictor for the PM_{10} concentration [3]. Since the measures directly influenced traffic intensity, the models in our study did not account for this factor.

Due to a three-month transitional period, in which violations were not fined, the measures did not continuously operate from one day to the other. Therefore, the transformation process is not reflected by the model.

References

1. Morfeld, P.; Spallek, M.; Groneberg, D. Zur wirksamkeit von umweltzonen: Design einer studie zur ermittlung der schadstoffkonzentrationsänderung für staubpartikel (PM_{10}) und andere größen durch einführung von umweltzonen in 20 deutschen städten (in German). *Zentralblatt Arbeitsmedizin Arbeitsschutz Ergonomie* **2011**, *61*, 138–165.
2. Vickers, A.J.; Altman, D.G. Statistics notes: Analyzing controlled trials with baseline and follow up measurements. *BMJ* **2001**, *323*, 1123–1124.
3. Fischer, P.H.; Hoek, G.; van Reeuwijk, H.; Briggs, D.J.; Lebret, E.; van Wijnen, J.H.; Kingham, S.; Elliott, P.E. Traffic-related differences in outdoor and indoor concentrations of particles and volatile organic compounds in Amsterdam. *Atmos. Environ.* **2000**, *34*, 3713–3722.

© 2014 by the authors; licensee MDPI, Basel, Switzerland. This article is an open access article distributed under the terms and conditions of the Creative Commons Attribution license (http://creativecommons.org/licenses/by/3.0/).
